# The multifaceted roles of receptor tyrosine pseudokinases in cellular signalling

**DOI:** 10.1042/BST20260770

**Published:** 2026-06-10

**Authors:** Emily C. Park, Andrew P. Thompson, James M. Murphy, Lung-Yu Liang, Isabelle S. Lucet

**Affiliations:** 1 Walter and Eliza Hall Institute of Medical Research, Parkville, VIC 3052, Australia; 2 Department of Medical Biology, University of Melbourne, Parkville, VIC 3052, Australia; 3 Centre for Cryo-electron Microscopy of Membrane Proteins, Monash Institute of Pharmaceutical Sciences, Monash University, Parkville, VIC 3052, Australia; 4 Drug Discovery Biology, Monash Institute of Pharmaceutical Sciences, Monash University, Parkville, VIC 3052, Australia; 5 Department of Molecular Sociology, Max Planck Institute of Biophysics, Frankfurt am Main 60438, Germany

**Keywords:** cellular communication, cellular signalling, pseudoenzyme, pseudokinases, Receptor tyrosine kinase

## Abstract

Receptor tyrosine pseudokinases comprise approximately 10% of the receptor tyrosine kinase family and lack phosphotransferase activity due to substitutions of essential catalytic residues within their kinase folds. This review critically examines the eight human receptor tyrosine pseudokinases: EphA10, EphB6, HER3, PTK7, ROR1, ROR2, RYK, and STYK1. By reconciling recent structural and functional data, we highlight the diverse non-catalytic mechanisms these pseudokinases employ in cellular communication. This comprehensive analysis provides insights into non-canonical signalling pathways and highlights potential for therapeutic opportunities in diseases associated with pseudokinase dysfunction.

## Introduction

Receptor tyrosine kinases are a major class of cell surface proteins that regulate cell proliferation, differentiation, migration, and survival through intercellular communication [1]. Upon binding of cognate protein ligands, these receptors undergo reorientation, dimerisation or clustering at the plasma membrane, bringing their intracellular kinase domains into close proximity and enabling trans-activation and initiation of downstream signalling events. As in most kinase families, approximately 10% of the receptor tyrosine kinases are classified as pseudokinases and are predicted to lack phosphotransferase activity due to substitutions of conserved catalytic residues in their kinase fold [2]. The emergence of pseudokinases has been attributed to gene duplication events, which can relieve selective pressures on the paralogues to maintain active site geometry [3,4]. This has allowed pseudokinases to evolve novel and specialised roles in the same signalling pathways as their active progenitors. While the signalling mechanisms of canonical receptor tyrosine kinases are increasingly well characterised, those dictated by pseudokinase members are understudied. In this review, we distil the current structural and functional understanding of the eight human receptor tyrosine pseudokinases—EphA10, EphB6, HER3, PTK7, ROR1, ROR2, RYK, and STYK1 (Table 1)—and highlight the diverse non-catalytic signalling mechanisms of these receptors in normal physiology and disease.

**Table 1:** List of the eight human receptor tyrosine pseudokinases and their known roles in cellular signalling

UniProt ID	Receptor name(s)	Acronym(s)	Subfamily	Size (AA)	Ligands	Signalling pathways	Cellular function	Tissue expression	Disease associations	Reference
Q5JZY3	Erythropoietin-producing hepatocellular carcinoma type-A receptor 10	EphA10	Eph	1008	Ephrin (A-class)	MAPK/ERK	Tissue patterning	Low in adult tissues, except male reproductive tissues	Breast, prostate, lung	[61]
O15197	Erythropoietin-producing hepatocellular carcinoma type-B receptor 6	EphB6	Eph	1021	Ephrin (B-class)	Rac1/RhoA GTPases	Tissue patterning	Ubiquitous	Breast, colorectal	[105]
P21860	Human epidermal growth factor receptor 3/Erythroblastic leukaemia viral oncogene homolog 3	HER3/ERBB3	HER	1342	Neuregulin	PI3K/Akt	Proliferation and survival	Ubiquitous	Various, particularly breast	[106]
Q13308	Protein tyrosine kinase 7/Colon Carcinoma Kinase 4	PTK7/CCK4	PTK7	1070	Wnt	Wnt/PCP, Wnt/β-catenin	Embryogenesis, neural tube closure	Low in adult tissues, except female reproductive tissues	Neural tube defects, various cancers	[107]
Q01973	Receptor tyrosine kinase-like orphan receptor 1	ROR1	ROR	934	Wnt	Wnt/PCP	Embryogenesis	Low in adult tissues	Blood	[108]
Q01974	Receptor tyrosine kinase-like orphan receptor 2	ROR2	ROR	943	Wnt	Wnt/PCP	Embryogenesis	Low in adult tissues	Pancreatic	[108]
P34925	Receptor like tyrosine kinase	RYK	RYK	607	Wnt, WIF	Wnt/PCP	Embryogenesis, axon guidance	Ubiquitous	Gastric	[109]
Q6J9G0	Serine/threonine/tyrosine kinase 1/Novel oncogene with kinase domain	STYK1/NOK	STYK	422	Unknown	PI3K/Akt, MAPK/ERK	Immune cell migration	Low in adult tissues	Lung, liver, blood	[110]

The receptor tyrosine pseudokinases EphA10, EphB6, and HER3 share homology with kinase-active counterparts in their cognate ligands and signalling pathways. PTK7, ROR1, ROR2, and RYK are members of pseudokinase-only subfamilies and act as co-receptors of the Wnt signalling pathway. STYK1 is a highly unconventional receptor tyrosine pseudokinase owing to a diminished ectodomain sequence, with much still unknown about its function.

## Domain organisation of receptor tyrosine pseudokinases and their ligands

**Figure 1:**
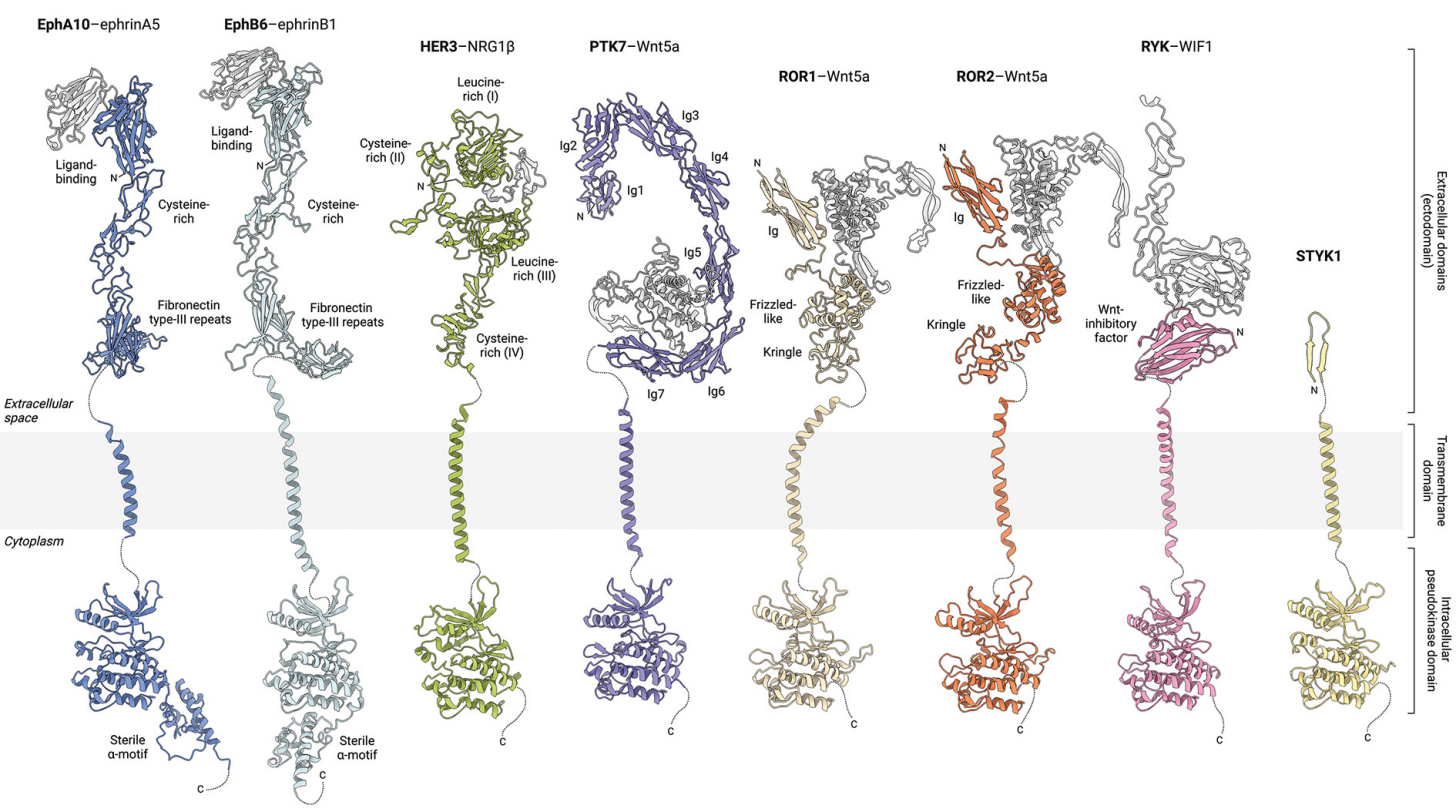
Domain organisation of the receptor tyrosine pseudokinases (coloured) and their ligands (grey) Domain organisation of the eight human receptor tyrosine pseudokinases closely resembles that of the kinase-active counterparts. Structural and functional analysis of these receptors can be divided into three key sections—the extracellular domains, transmembrane domain, and intracellular domains—which serve to transduce cellular signals across the membrane. Individual extracellular and accessory intracellular domains are labelled. All 3D structures shown are based on AlphaFold3 [125] models with unstructured regions shown as dotted lines. Current available structural information is summarised in Table 3.

Receptor tyrosine pseudokinases, like their kinase-active counterparts, are comprised of an extracellular ligand-binding region connected by a single-pass transmembrane domain to an intracellular region which harbours the pseudokinase domain (Figure 1). This structural organisation reflects an ability to transmit external signals into cellular responses [1,5]. In canonical receptor tyrosine kinase signalling, ligand binding to the extracellular region induces conformational change leading to receptor dimerisation and activation of the intracellular tyrosine kinase domain. Cross-phosphorylation of the juxtaposed receptor monomers creates binding sites for downstream adaptors and effector proteins that initiate diverse signalling cascades. Although receptor tyrosine pseudokinases lack intrinsic catalytic activities, their conserved domain organisation enables participation in signal transduction through non-catalytic mechanisms, including ligand recognition, receptor homo- and heterodimerisation, and engagement with downstream signalling proteins [5].

Beginning at the extracellular region (or ectodomain), receptor tyrosine pseudokinases exhibit diverse and modular structures reflective of their distinct subfamilies. For example, the ectodomain of HER3 is comprised of alternating leucine-rich (I and III) and cysteine-rich (II and IV) domains. Like active HER family receptors, ligands such as epidermal growth factor (EGF) and neuregulin (NRG) bind with high affinity to a groove formed by domains I and III [6]. By comparison, the ectodomains of Eph receptors are comprised of a ligand-binding domain followed by a cysteine-rich domain and two fibronectin type-III domains. In active Eph receptors, binding of ligands called ephrins to a high-affinity pocket formed by the ligand-binding domain is driven by the insertion of a flexible and solvent-exposed region of the ephrin known as the G–H loop [7]. Structural details of receptor-ligand interactions have not been experimentally described for EphA10 or EphB6; however, a crystal structure of the EphB6 ectodomain closely resembles those reported of other Eph receptors [8]. Interestingly, the ligand-binding domain of EphB6 has an insertion of 11 serine residues within the J–K loop located within the ephrin ligand-binding site that is not present in any other Eph receptor. Because the J–K loop is part of the receptor-ligand interface, it was suggested that EphB6 may have lower affinity for ligands due to interference by the polyserine insert, which is predicted to be highly flexible [8].

While EphA10, EphB6, and HER3 share homology with kinase-active receptors and their cognate ligands, PTK7, ROR1, ROR2, and RYK are members of pseudokinase-only subfamilies. These receptors were previously described as orphan receptors, meaning their ligands were unknown, but have all emerged to play roles in Wnt signalling pathways since their initial classification [7,9,10]. For example, ROR1 binds non-canonical Wnt ligands such as Wnt5a through a hydrophobic pocket in its cysteine-rich domain [7]. This hydrophobic pocket is conserved for binding lipids and lipid-modified ligands in many Wnt receptor proteins such as the Frizzled receptor family [7]. Similarly, the Wnt-inhibitory factor (WIF) domain of RYK interacts with non-canonical Wnt ligands and WIFs directly through lipid modifications [9]. In both cases, ROR1 and RYK antagonise canonical Wnt/β-catenin signalling pathways. However, recent structural characterisation of ROR2 suggests a distinct mechanism of Wnt ligand recognition. The cysteine-rich domain of ROR2 has evolved an additional helical structure that blocks exposure of any possible hydrophobic, lipid-binding pocket [11]. It may be possible that Wnt ligands bind alternative sites or require a conformational rearrangement that is not yet described. Besides the cysteine-rich domain, ROR receptors harbour an immunoglobulin domain and a Kringle domain thought to drive receptor dimerisation [12]. By comparison, the ectodomain of PTK7 is comprised of seven immunoglobulin domains and interacts with both canonical and non-canonical Wnt ligands [13]. Structural information for the PTK7 ectodomain has not yet been reported and the specific molecular interaction of this receptor with Wnt ligands remains unclear. In complete contrast with other receptor tyrosine pseudokinases, STYK1 only harbours a short extracellular segment of 22 amino acids with no reported ligands. The absence of a plausible ligand-binding ectodomain suggests that STYK1 may activate signalling cascades independent of external cues [14], although the prospect of activation via a co-receptor cannot be excluded.

The ectodomain of each receptor is connected to its intracellular region by a single-pass transmembrane domain with shared features [15]. Around 25–38 amino acids in length, this α-helical structure anchors each receptor through interfaces with the lipid bilayer. More recently, dimerisation of the transmembrane domains has been shown to play a critical role in signal transduction across the membrane (described in a later section).

Intracellularly, these receptors possess pseudokinase domains with diverse signalling capacities, despite variations in critical motifs that would be expected to impair or eliminate their kinase activity. These include contributions from a VAIK motif in the β3 strand, an HRD motif in the catalytic loop, and a DFG motif in the activation loop to coordinate ATP for phosphoryl group transfer to a substrate (Table 2). Many of the receptor tyrosine pseudokinases have retained conformational flexibility and ATP-binding capacity of their pseudokinase domains, allowing them to engage in allosteric signalling mechanisms [2,5,16–18]. For example, the pseudokinase domain of HER3 functions as an allosteric activator of HER family kinases such as HER2 and epidermal growth factor receptor (EGFR), where ATP binding is suggested to play a role in conformational stabilisation [19,20]. The pseudokinase domains of EphA10 and EphB6 also retain ATP-binding sites and can bind both type I and type II kinase inhibitors, which stabilise the active and inactive states of canonical kinases, respectively [42,43]. The propensity of small molecules to engage the ATP-binding pocket and stabilise receptor tyrosine pseudokinases raises the possibility that each has retained the conformational switch-based regulatory mechanisms established for active kinases [1], which may be used to control interactions with binding partners.

**Table 2:** Divergence of canonical catalytic residues in the pseudokinase domain of receptor tyrosine pseudokinases

Conserved motifs	Canonical RTK	EphA10	EphB6	HER3	PTK7	ROR1	ROR2	RYK	STYK1
Glycine-rich loop	**G**X**G**XX**G**	GXGXXG	GXGXXG	GXGXXG	GXSXXG	GXCXXG	GXDXXG	QXGXXG	CXGXXG
β3-strand	VAI**K**	VAVH	VAIQ	VCIK	VLVK	VAIK	VAIK	AFVK	VILK
αC-helix	E	E	R	H	E	E	E	E	R
Catalytic loop	HR**D**XXXX**N**	HRGXXXXH	HRSXXXXS	HRNXXXXN	HKDXXXXN	HKDXXXXN	HKDXXXXN	HKDXXXXN	HKDXXXXN
Activation loop	**D**FG	GFG	RLG	DFG	ALG	DLG	DLG	DNA	GLG
ATP-binding site	Accessible	Accessible	Accessible	Accessible	Occluded	Occluded	Occluded	Occluded	Unknown

Essential residues for catalysis in the kinase domain of a canonical receptor tyrosine kinase (RTK) are bolded. Corresponding residues that differ in the pseudokinase domain of receptor tyrosine pseudokinases are underlined. Active site occlusion is defined by structural and/or ATP binding studies. X represents any amino acid.

**Table 3:** Available structural information of the human receptor tyrosine pseudokinases

Receptor	Domains	Method	PDB ID	Reference
EphB6	Ectodomain	X-ray diffraction	7K7J	[8]
HER3	Ectodomain	X-ray diffraction	1M6B	[111]
HER3	Ectodomain	X-ray diffraction	3P0Y, 3P11	[112]
HER3	Ectodomain	X-ray diffraction	4LEO	[113]
HER3	Ectodomain	X-ray diffraction	4P59	[114]
HER3	Ectodomain	X-ray diffraction	5CUS	[115]
HER3	Ectodomain	X-ray diffraction	5O4G, 5O4O, 5O7P, 5O7O	[116]
HER3	Ectodomain	X-ray diffraction	7BHE, 7BHF	[117]
HER3	Ectodomain	X-ray diffraction	7D85	[118]
HER3	Ectodomain	Electron microscopy	7MN5, 7MN6, 7MN8	[6]
HER3	Ectodomain	Electron microscopy	8YRY	[119]
HER3	Ectodomain	X-ray diffraction	9I1Q	[120]
HER3	Transmembrane	Solution NMR	2L9U	[34]
HER3	Transmembrane	Solution NMR	2N2A	[33]
HER3	Kinase	X-ray diffraction	3KEX	[19]
HER3	Kinase	X-ray diffraction	3LMG	[20]
HER3	Kinase	X-ray diffraction	4RIW, 4RIX, 4RIY	[40]
HER3	Kinase	X-ray diffraction	6OP9	[66]
PTK7	Kinase	X-ray diffraction	6VG3	[2]
ROR1	Kringle	Solution NMR	5Z55	[121]
ROR1	Kringle	X-ray diffraction	6BA5, 6BAN	[122]
ROR1	Kringle	X-ray diffraction	7TNG	[123]
ROR1	Kinase	X-ray diffraction	6TU9	[2]
ROR2	Kringle	X-ray diffraction	6OSH, 6OSN, 6OSV	[124]
ROR2	Cysteine-rich, Kringle	X-ray diffraction	9FSE	[11]
ROR2	Kinase	X-ray diffraction	4GT4	[22]
RYK	Kinase	X-ray diffraction	6TUA	[2]

No experimentally determined structures are currently available for EphA10 or STYK1.

Like EphA10 and EphB6, the conformational flexibility of ROR1 and ROR2 can be modulated by kinase inhibitors, even though their ATP-binding sites are occluded [2,21,22]. In these receptors, as well as PTK7 and RYK, divergence from canonical kinase residues results in atypical ATP-binding site architecture that differs from active kinases (Table 2). For example, the ATP-binding site of PTK7 is occluded by side chains from a leucine (L949) in its activation loop and a tyrosine (Y877) in its hinge region between the N- and C-terminal lobes [2,23]. Interestingly, the activation-loop conformation of PTK7, ROR1, ROR2, and RYK each closely resemble the autoinhibited conformation of insulin family receptors, which share common ancestors [2,21,22]. Whether the STYK1 pseudokinase domain binds ATP is yet to be formally examined, although introduction of a K147R mutation in the VAIK motif, a conserved ATP binding motif, was found to compromise interactions with components of downstream signalling complexes [24]. Additionally, the absence of the canonical DFG motif in the activation loop (Table 2) is expected to preclude Mg^2+^ cofactor binding and therefore diminish any potential for catalytic activity. Outside their pseudokinase domains, several additional elements contribute to the intracellular signalling functions of receptor tyrosine pseudokinases, including phosphorylation sites, adaptor-binding motifs, and dimerisation domains (described in a later section) [5].

## Mechanisms of receptor dimerisation and clustering for signal transduction

**Figure 2:**
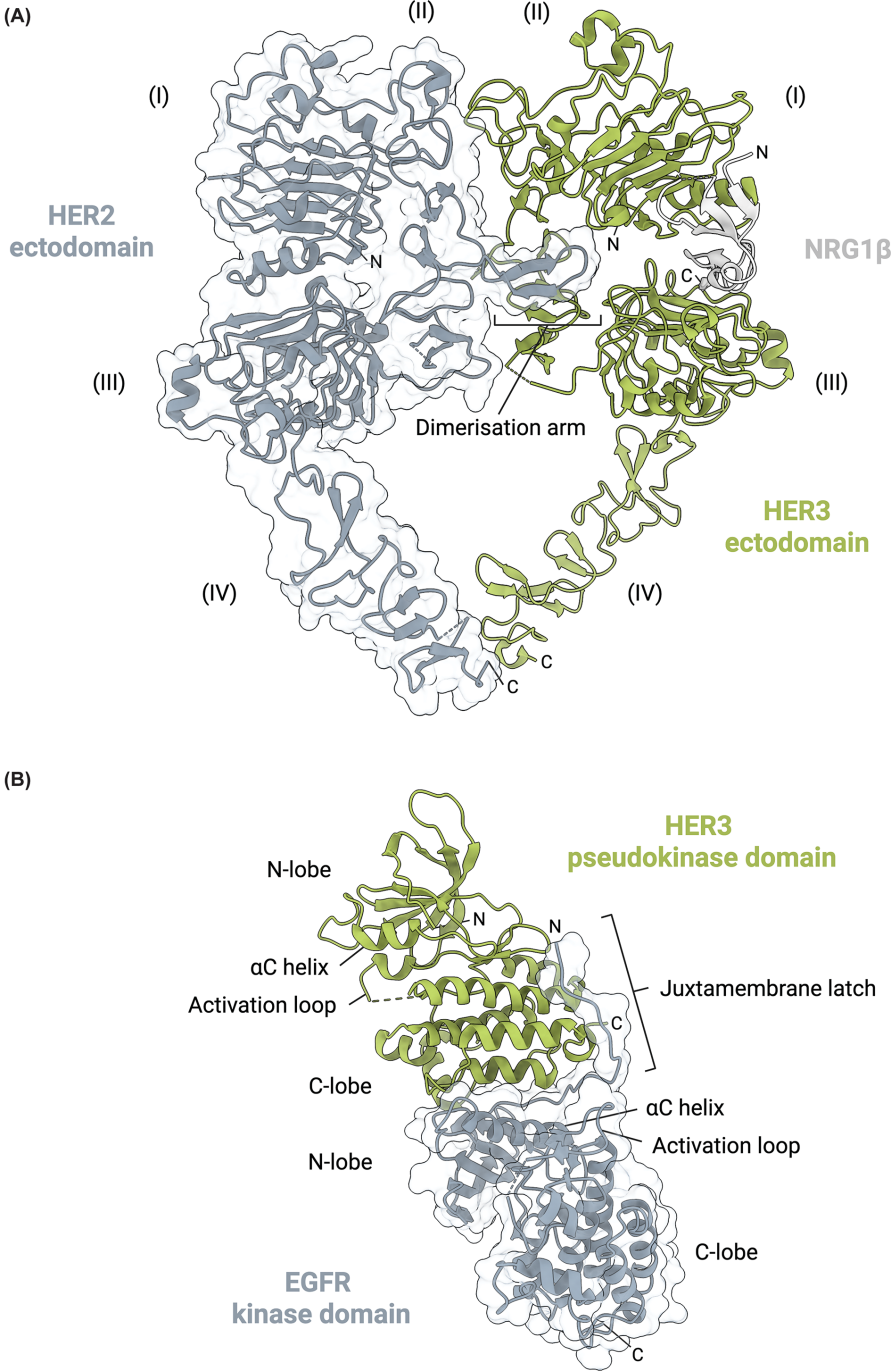
Allosteric activation of kinase-active epidermal growth factor receptors by receptor tyrosine pseudokinase HER3 (**A**) Cryo-electron microscopy structure of the HER2-HER3-NRG1β ectodomain complex (PDB: 7MN5). HER2–HER3 heterodimerisation is induced by binding of NRG1β between the leucine-rich domains of HER3. In its active state, the dimerisation arm of HER2 contacts a pocket formed by the leucine-rich domains of HER3. The reciprocal dimerisation arm of HER3 is unresolved. (**B**) Structure of the HER3–EGFR asymmetric (pseudo)kinase domain heterodimer (PDB: 4RIW). A C-terminal portion of the EGFR juxtamembrane region forms a stabilising latch between the N-terminal lobe of EGFR and C-terminal lobe of HER3.

**Figure 3:**
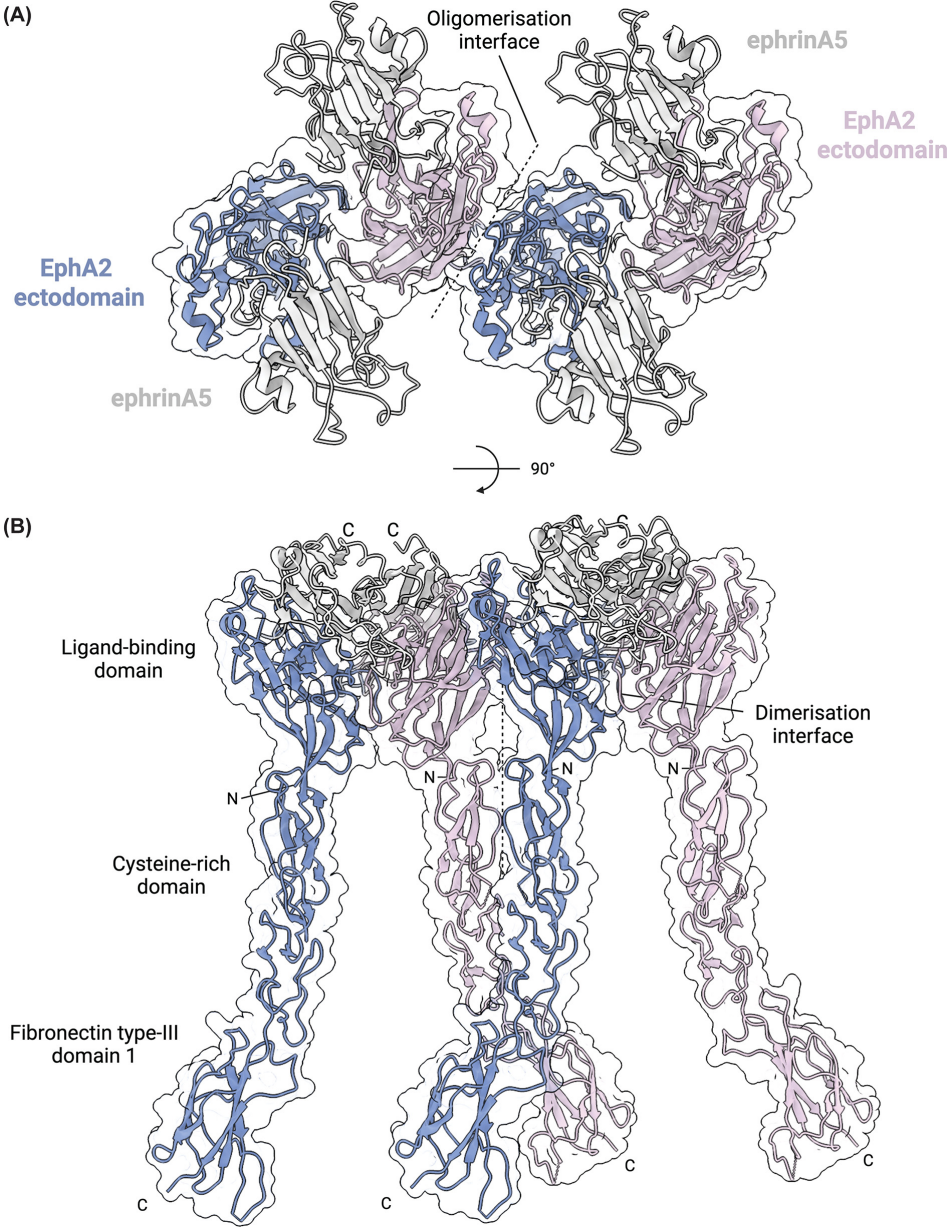
Ligand-induced dimerisation and clustering of Eph receptor (pseudo)kinases Crystal structure of the EphA2-ephrinA5 heterotetramer from (**A**) top and (**B**) side views (PDB: 3MX0). Receptor dimerisation is mediated by the ligand-binding domain at a distinct and lower-affinity site to ephrin binding. Crystal packing suggests two additional interfaces for receptor oligomerisation involving the ligand-binding and cysteine-rich domains. These interfaces have been observed in other Eph–ephrin assemblies, including ligand-independent Eph receptor dimers. Additional interfaces in the transmembrane and SAM domains are also suggested to regulate receptor dimerisation and clustering.

Receptor dimerisation is an essential element of signal transduction by canonical receptor tyrosine kinases [1]. Receptor dimerisation is induced by ligand binding and involves a series of low-affinity interfaces across the extracellular, transmembrane, and intracellular domains of neighbouring receptors within the plasma membrane. Notably, ligand binding promotes conformational changes to the receptor ectodomain to enable dimerisation that are minimal in some cases and substantial in others. In HER3, binding of growth factors induces a substantive conformational change in the ectodomain through a similar mechanism of the kinase-active HER family receptors [6]. This conformational change exposes a key structural feature from domain II called the dimerisation arm, which contacts a neighbouring receptor in a pocket formed by domains I and III (Figure 2) [6]. Prior to ligand binding, the dimerisation arm is buried by intramolecular interactions between domains II and IV that autoinhibit receptor dimerisation. Through this mechanism, HER3 forms heterodimers with kinase-active receptors such as HER2 and EGFR to allosterically regulate their activity but does not form homodimers [6]. Compared with HER3, binding of ephrins to Eph receptors induces a minimal conformational change; however, the homo- or heterodimerisation mechanisms of these receptors have only been described through crystal packing data (Figure 3) [25–30]. An additional unique mechanism of regulation has been proposed from crystal packing data of unliganded Eph receptor ectodomains, including EphB6, whereby the ligand-binding domain of one receptor binds to the fibronectin type III domain of a neighbouring receptor, but the biological relevance of this ‘head-to-tail’ interaction is still debated [8,30]. Evidence also supports both homo- and heterodimerisation of ROR family receptors induced by binding of Wnt5a [21]. Through co-immunoprecipitation experiments, it has been suggested that the Kringle domain is minimally required for ROR dimerisation; however, this result has not been supported by structural data [12]. Mechanisms of receptor dimerisation are yet to be established for PTK7, RYK, and STYK1.

More recently, transmembrane domain associations of receptor tyrosine kinases have emerged as key to understanding their mechanisms of signal transduction [31,32]. For example, nuclear magnetic resonance (NMR) experiments have identified two interfaces for transmembrane domain association at the N- and C-terminal ends of HER family receptors [33,34]. During receptor activation, a switch mechanism is proposed to vary the helix crossing angle of the dimerised transmembrane domains as well as the distance between each terminus. Importantly, this switch mechanism drives juxtaposition of the intracellular kinase domains and therefore offers an additional method of receptor regulation. Recent molecular dynamics simulations of transmembrane domain dimerisation in Eph receptors have proposed a similar switch mechanism [35–37]. These studies also investigate the importance of lipid compositions for regulating transmembrane domain dimerisation and interactions of the juxtamembrane and membrane proximal regions with the lipid bilayer. However, it should be noted that experimental study of full-length single-pass transmembrane receptors remains enormously challenging due to their inherent flexibility [38], and therefore NMR and molecular dynamics experiments may not present a complete picture. HER and Eph family receptors also share a conserved N-terminal glycine zipper motif (GXXXG) that is found in ∼50% of all transmembrane domains [39]. Although not described for receptor tyrosine pseudokinases, specific mutations to this and other motifs in the transmembrane domains have been shown to modulate the stability of receptor dimers and residency at the membrane [31]. Moreover, small peptides have been demonstrated to specifically recognise the transmembrane domain of single-pass transmembrane receptors and alter their configuration, providing a valuable tool for studying transmembrane domain associations and their regulatory role [15].

Despite lacking tyrosine kinase activity, the intracellular regions of receptor tyrosine pseudokinases retain key residues for phosphorylation by catalytically active receptor tyrosine kinases [2]. These trans-phosphorylation events create docking sites that recruit adaptors and signalling effectors containing Src homology 2 (SH2) or other phosphotyrosine-binding domains. Critically, dimerisation of the receptors’ extracellular and transmembrane domains juxtaposes the intracellular domains so that trans-phosphorylation can occur. For example, the pseudokinase domain of HER3 and the kinase domain of EGFR were shown to adopt an asymmetric dimer (Figure 2) [19,40]. This dimer is formed by interactions between the N-terminal lobe of the EGFR kinase domain and the C-terminal lobe of the HER3 pseudokinase domain with stabilisation by the unstructured juxtamembrane region of EGFR. Allosteric activation of the EGFR kinase domain catalyses the transfer of phosphate groups onto the C-terminal tail of HER3, recruiting phosphatidylinositol 3 kinase (PI3K) and initiating the PI3K/Akt signalling pathway. By comparison, juxtaposition of the Eph receptor kinase domains is stabilised by dimerisation of the C-terminal sterile α-motif (SAM) domains [41,42]. Two conserved phosphosites (JX1 and JX2) on the juxtamembrane region of Eph receptors provide binding sites for SH2 domain-containing proteins. In EphB6, the phosphosites JX1 and JX2 are substrates for phosphorylation by EphB4 and recruit signalling proteins such as Abl, Src, and Vav3 [43]. However, EphA10 lacks these phosphosites in its juxtamembrane region, and the identities of downstream effectors of EphA10 signalling and how they engage remain to be characterised.

In addition to phosphosites, the receptor tyrosine pseudokinases also retain motifs for phospholipid binding and other protein interaction domains within their intracellular regions [44–46]. For example, the proline-rich region on the C-terminal tail of ROR receptors contains multiple binding sites for SH3 domain-containing proteins, including guanine nucleotide exchange factors [12]. Heterodimerisation of ROR1 and ROR2 induced by Wnt5a recruits guanine nucleotide exchange factors and activates signalling pathways of Rho GTPases. In this manner, receptor tyrosine pseudokinases serve as scaffolds and facilitate the dynamic assembly of multimeric signalling hubs.

Signal transduction events by some receptor tyrosine pseudokinases can also involve the formation of oligomers and higher-order clusters at the plasma membrane. Uniquely, the size of clusters (ranging from hundreds of nanometres up to micrometres) and the corresponding level of signal amplification can be directly correlated with the receptors and ligands involved. This phenomenon has been observed through live-cell imaging of EphB6, HER3, PTK7, and ROR2, appearing as puncta at the cell surface by fluorescence microscopy [47–50]. In each case, the receptors did not form clusters until induced by ligand binding, although it may be possible that non-liganded receptors are recruited into clusters. For example, the extent of HER3 clustering was shown to be dependent on stimulation by neuregulins or epidermal growth factors, which drive distinct mechanisms of activation [47]. Further investigation into the architecture and compositions of these clusters will be important for understanding the diverse signalling capabilities of receptor tyrosine pseudokinases. One of the major challenges with studying these clusters is that introduction of fluorescent tags and overexpression cell culture systems may contribute to receptor clustering [48,50]. Therefore, a detailed understanding of endogenous signalling awaits the development of suitable fluorescent probes.

## Intercellular and intracellular signalling pathways coordinate cell behaviours

From their position at the plasma membrane, the receptor tyrosine pseudokinases can regulate cell behaviours through multiple non-catalytic signalling modalities such as dimerisation, proteolytic cleavage, isoforms, and receptor cross-talk. For instance, the Eph receptors, including EphA10 and EphB6, are unique from other receptor tyrosine kinases in that their ephrin ligands are membrane-bound. This feature enables contact-dependent and bidirectional signalling between Eph- and ephrin-presenting cells, with important implications in cell adhesion and migration [51]. Moreover, the composition of Eph–ephrin co-clusters has been shown to dramatically alter the extent of clustering and their cellular responses. Recently, EphB6 was shown to form large co-clusters with ephrinB1 that promote stable cell adhesion and were resistant to endocytosis, unlike those formed by a kinase-active counterpart [48]. These results suggested a role for EphB6 as a cellular glue; however, it is likely that different receptor-ligand pairings in a specific cellular context will lead to distinct cellular responses.

While EphA10 and EphB6 rely on their membrane-bound ligands to form cell–cell contacts, PTK7 facilitates cell adhesion through receptor dimerisation alone [13]. PTK7 is dramatically localised to sites of cell-cell contact and forms homotypic interactions between its extracellular immunoglobulin-like domains in trans. Specifically, PTK7 localises at the adherens junctions of migrating epithelial cells and associates with other adhesion proteins such as E-cadherin and β-catenin to further stabilise cell–cell junctions [52]. Interestingly, the size of PTK7 clusters appears to be influenced by both the abundance of cholesterol in the lipid bilayer and the degree of PTK7 glycosylation [50]. This suggests that plasma membrane fluidity and post-translational modifications of the PTK7 ectodomain are important for its regulation.

Emerging evidence suggests that cell–cell interactions by receptor tyrosine pseudokinases could also be mediated by specialised membrane protrusions called cytonemes in some cell types. These thin, actin-based structures extend from cells to facilitate long-distance communication and exchange of signalling molecules [53]. Cytoneme-mediated signalling has been demonstrated for ROR2 in the epiblast and fibroblast cells of zebrafish embryos [54]. In this case, ROR2–Wnt5b complexes were loaded onto cytonemes for transfer from the receptor-presenting cell to a target cell via endocytosis. Wnt/planar cell polarity (PCP) signalling is then initiated within the target cell, irrespective of its expression of ROR2 or other Wnt receptors. Recently, the formation of tubular membrane structures between EphB6- and ephrinB1-presenting cells was shown to be induced by co-clustering of EphB6 and ephrinB1 at junctions of metastatic breast cancer cells [48]. These structures are distinct from cytonemes but similarly facilitate contact-dependent signalling between cells. EphB6-ephrinB1 clusters were shown to migrate along the tubular structures, some of which became endocytosed by the ligand-presenting cells [48].

Ectodomain shedding refers to the proteolytic cleavage of cell surface proteins leading to release of their extracellular domains as soluble components. This post-translational process is used to dynamically regulate expression and functionality of membrane proteins [55]. Amongst the receptor tyrosine pseudokinases, ectodomain shedding of PTK7 is the best understood [13]. PTK7 is targeted by membrane type-1 matrix metalloproteinase (MT1-MMP), a transmembrane enzyme known for extracellular matrix remodelling and regulation of cell migration through its proteolytic activities [56]. MT1-MMP cleaves PTK7 at residue L622 within the seventh immunoglobulin-like domain. Recently, the cleaved ectodomain of PTK7 has been shown to dimerise with full-length PTK7 at the membrane and promote cell proliferation and migration through Wnt signalling [57]. Following release of the PTK7 ectodomain, the remaining membrane-bound components undergo two further cleavages by a disintegrin and metalloproteinase 17 (ADAM17) and γ-secretase [58]. The cleaved, intracellular fragment translocates to the nucleus and promotes cell proliferation and migration through transcriptional regulation. Similar processes have been suggested for both ROR1 and RYK [9].

In addition to proteolytic cleavage, many of the receptor tyrosine pseudokinases exist as soluble isoforms derived from alternative splicing events [55]. Importantly, such isoforms tend to possess distinct interaction networks, expression patterns, and disease associations. For each receptor tyrosine pseudokinase, up to six isoforms have been described in the UniProt database [59]; however, the biological relevance of these isoforms has been assessed. For example, HER3 exists as two alternative isoforms called p45 and p85 soluble HER3, which only contain parts of the ectodomains [60]. p85-soluble HER3 has been described as a potent negative regulator of HER2, HER3, and HER4 activity. Specifically, p85-soluble HER3 binds to growth factor neuregulin and inhibits neuregulin-mediated activation. Similarly, EphA10 exists as an isoform called EphA10s that comprises only the ligand-binding and cysteine-rich domains, driven by splicing of mRNA at exon 3 on chromosome locus 1p34.3 [61]. Proposed to counteract the oncogenic activity of full-length EphA10 (described further below), down-regulation of EphA10s may play a role in promoting malignant transformation [62]. mRNA transcripts of PTK7 splice variants have been identified with deletions of the first, sixth, or seventh immunoglobulin domains from exons 8–16 encoded by chromosome locus 6p12.2–21.2 [63]. Expression patterns of the mRNA transcripts differ between cancer cell lines and likely contribute to functional diversity of PTK7 signalling.

Trans-phosphorylation of receptor tyrosine pseudokinases can also occur through cross-talk with receptor tyrosine kinases outside of the receptor’s family, as well as soluble kinases within the cytoplasm. The signalling pathways that are regulated through receptor cross-talk are specific to the roles of each kinase-active partner. For example, ROR2 has been reported to mediate non-canonical Wnt signalling through dimerisation with Frizzled receptors via its cysteine-rich domain [11]. Additionally, STYK1 has been proposed to mediate STAT3/STAT5 signalling through dimerisation with EGFR via its intracellular juxtamembrane and kinase domains [14]. However, transphosphorylation events between the kinase-active and -inactive receptors in these examples are yet to be established. Cross-talk between EGFR and EphB6 has also been predicted through genetic and proteomic screening of cancer-related interaction networks, supported by positive correlation of expression levels in several cancer types [64]. Interestingly, co-expression of EphB6 in an EGFR-positive cancer cell line was shown to increase EGF-induced phosphorylation of EGFR, implying that EphB6 interacts with EGF-induced EGFR dimers. Combined targeting may offer an effective treatment in tumours where both receptors are expressed.

## Dysregulation of cellular signalling in cancer and other diseases and therapeutic strategies

HER3 is the most widely characterised receptor tyrosine pseudokinase, owing to its prevalent role in cancer progression [65]. The oncogenic activity of HER3 stems from its role as an allosteric activator of other HER family receptors and has been associated with resistance to kinase inhibitor therapies by reactivating their active counterparts [66]. This adaptive signalling ability makes HER3 a critical target for cancer therapies, particularly in overcoming resistance to treatments targeting other HER family members. Interestingly, concurrent missense mutations in the kinase domain of HER2 and pseudokinase domain of HER3 have been demonstrated to promote oncogenic signalling of the heterodimeric complex [67]. Overexpression of HER3 has also been observed in multiple types of cancer such as breast (10.0%) [68], ovarian (12.4%) [69], and non-small cell lung cancer (NSCLC) (25%) [70]. Several antibody-based therapeutics targeting the ectodomain of HER3 are currently in development [65].

Dysregulation of the Eph receptor pseudokinases, EphA10 and EphB6, is strongly linked to the development and progression of multiple cancer types [71]. Interestingly, EphA10 and EphB6 are proposed to serve opposite roles, with oncogenic and tumour-suppressive functions, respectively. EphA10 is normally expressed at undetectable levels, except in male reproductive tissues [61], but is differentially expressed in prostate cancer [72], breast cancer [62], NSCLC [73], lung adenocarcinoma [74], and pancreatic ductal adenocarcinoma [75]. Interestingly, an EphA10 isoform (EphA10s) containing only the ligand-binding and cysteine-rich domains has been found to counteract the tumour-promoting activity of full-length EphA10 in breast cancer models [62]. Targeting of EphA10 with monomethyl auristatin E antibody-drug conjugates has demonstrated strong cytotoxic effects in EphA10-positive breast cancer cells [76]. In comparison, EphB6 is expressed in all normal tissue types and is down-regulated in several malignancies, including breast and colorectal cancers [77–79].

PTK7, ROR1, ROR2, and RYK each play essential roles in embryogenesis [80]. They exhibit a distinct pattern of expression, characterised by high levels of expression during embryonic development, followed by down-regulation in adult tissues [81]. PTK7 is most known for its role in Wnt signalling and the planar cell polarity pathway, and is required in critical neural development processes, such as neural tube closure [82]. Although rare, missense variants in *PTK7* are suggested to contribute to the risk of neural tube defects and congenital malformations such as spina bifida [82,83]. Additionally, overexpression of PTK7 is associated with onset and progression of several solid and haematological malignancies with poor prognosis [23]. Several targeted therapies aimed at inhibiting PTK7 to induce antitumour effects are under preclinical and clinical investigation and were recently reviewed in detail [84]. Notably, an anti-PTK7 antibody-drug conjugate cofetuzumab pelidotin (PF-06647020) has been investigated in Phase I clinical trials in patients with advanced solid tumours, demonstrating a tolerable safety profile and preliminary anti-tumour activity (NCT02222922, NCT04189614) [85,86].

Like PTK7, up-regulation of the ROR receptors in adult pathologies has been observed in multiple cancer types and is correlated with poor prognosis, including chronic lymphocytic leukaemia (CLL) and colon cancer [87,88]. ROR1, in particular, has been presented as a promising biomarker for CLL and potential therapeutic target due to its selective expression in 95% of neoplastic B cells [89]. Cirmtuzumab is an anti-ROR1 monoclonal antibody which targets the ROR1 ectodomain and was investigated in Phase I clinical trials (NCT02222688). Cirmtuzumab treatment in CLL patients was shown to reduce activation of Rho GTPases and haematopoietic lineage-specific protein 1 (HS1) through inhibition of ROR1 signalling and reduce gene signatures of CLL stemness [89]. Meanwhile, ROR2 has been reported to drive development of pancreatic ductal adenocarcinoma and resistance to inhibitors of Kirsten rat sarcoma viral oncogene homologue (KRAS) [90]. Preclinical development of antibody-based therapeutics targeting ROR receptors is ongoing, most recently including a ROR1-specific antibody with antibody-dependent cellular cytotoxicity activity (PBA-045) and a ROR2-specific antibody-drug conjugate (BA3021) [91,92].

RYK plays a specific role in embryonic development of the nervous system, including cell polarity and migration processes required for axon guidance [93,94]. However, much remains unknown about the signalling mechanisms of RYK in non-canonical Wnt pathways. Knockdown of RYK has been reported to suppress the proliferation and invasion of gastric cancer cells [95,96], suggesting an oncogenic role. Recent bioinformatic analysis of expression in various cancers, prognostic significance, and involvement in cancer-related signalling pathways provides insights into the role of RYK in cancer biology [97].

Lastly, STYK1 is the most recently discovered receptor tyrosine pseudokinase. While much is still unknown about this receptor, it has been reported to activate several critical signalling pathways, including the PI3K/Akt and mitogen-activated protein kinase (MAPK) pathways which promote cell proliferation and survival. Overexpression of STYK1 is correlated with poor prognosis in numerous types of cancer, including hepatocellular carcinoma [98], pancreatic ductal adenocarcinoma [99,100], NSCLC [101], breast cancer [102], and leukaemia [103]. Interestingly, STYK1 was proposed to play a contrasting role in glioma, where it exhibited an anti-tumour effect [104]. In this context, STYK1 is primarily expressed in natural killer cells and was implicated in the anti-tumour response. There are currently no reported compounds targeting STYK1.

## Concluding remarks

Since their discovery in the 1990s and early 2000s, extensive evidence has emerged supporting the role of receptor tyrosine pseudokinases in cellular signalling and disease processes, although our molecular-level knowledge is limited. A comprehensive mechanistic understanding of signal transduction by receptor tyrosine pseudokinases and their regulation of kinase-active receptors will unravel biologically relevant interaction networks and guide the effective design of therapeutic strategies in human malignancies.

## Perspectives

- Receptor tyrosine pseudokinases are critical regulators of cellular communication. They challenge traditional understanding of kinase signalling and reveal the complexity and diversity of cellular signalling networks driven by non-catalytic mechanisms.
- Recent studies have uncovered remarkable diversity in receptor tyrosine pseudokinase signalling. Their unique structural features and unconventional signalling mechanisms, from allosteric regulation to scaffolding functions, have revealed novel therapeutic opportunities, challenging the notion that kinase activity is essential for druggability.
- The success of TYK2 pseudokinase inhibitor deucravacitinib in the clinic demonstrates the feasibility of targeting pseudokinases therapeutically. This breakthrough should encourage exploration of similar approaches for receptor tyrosine pseudokinases, potentially leading to novel treatments for associated diseases.

## Open Access

Open access for this article was enabled by the participation of University of Melbourne in an all-inclusive Read & Publish agreement with Portland Press and the Biochemical Society under an agreement with CAUL.

## Abbreviations

ADAM: A disintegrin and metalloproteinase
CAR: Chimeric antigen receptor
CLL: Chronic lymphatic leukaemia
EGFR: Epidermal growth factor receptor
Eph: Erythropoietin-producing human hepatocellular
EphA10s: EphA10 isoform
FGFR: Fibroblast growth factor receptor
HER: Human epidermal growth factor receptor
HS1: Haematopoietic lineage specific protein 1
KRAS: Kirsten rat sarcoma viral oncogene homolog
MAPK: Mitogen activated protein kinase
MT1-MMP: Membrane type-1 matrix metalloproteinase
NRG: Neuregulin
NSCLC: Non-small cell lung cancer
PCP: Planar cell polarity
PI3K: Phosphatidylinositol 3 kinase
PTK: Protein tyrosine kinase
ROR: Receptor tyrosine kinase-like orphan receptor
RYK: Receptor-like tyrosine kinase
SAM: Sterile α-motif
SH: Src homology
STAT: Signal transducer and activator of transcription
STYK: Serine/threonine/tyrosine kinase
WIF: Wnt inhibitor factor
